# COVID-19 and Cancer Diseases—The Potential of *Coriolus versicolor* Mushroom to Combat Global Health Challenges

**DOI:** 10.3390/ijms24054864

**Published:** 2023-03-02

**Authors:** Tomasz Jędrzejewski, Małgorzata Pawlikowska, Justyna Sobocińska, Sylwia Wrotek

**Affiliations:** Department of Immunology, Faculty of Biological and Veterinary Sciences, Nicolaus Copernicus University, 1 Lwowska Str., 87-100 Torun, Poland

**Keywords:** *Coriolus versicolor*, cancer, COVID-19, immunomodulation, angiogenesis, inflammation, fever

## Abstract

*Coriolus versicolor* (CV) is a common species from the *Polyporaceae* family that has been used in traditional Chinese herbal medicine for over 2000 years. Among well-described and most active compounds identified in CV are polysaccharopeptides, such as polysaccharide peptide (PSP) and Polysaccharide-K (PSK, krestin), which, in some countries, are already used as an adjuvant agent in cancer therapy. In this paper, research advances in the field of anti-cancer and anti-viral action of CV are analyzed. The results of data obtained in in vitro and in vivo studies using animal models as well as in clinical research trials have been discussed. The present update provides a brief overview regarding the immunomodulatory effects of CV. A particular focus has been given to the mechanisms of direct effects of CV on cancer cells and angiogenesis. A potential use of CV compounds in anti-viral treatment, including therapy against COVID-19 disease, has also been analyzed based on the most recent literature. Additionally, the significance of fever in viral infection and cancer has been debated, providing evidence that CV affects this phenomenon.

## 1. Introduction

Natural products have played a vital role in health care since ancient times. In academic medicine, there are many examples of drugs (including chemotherapeutic agents) that originated from plants and mushrooms, e.g., topotecan [1], etoposide, teniposide [2], docetaxel, and paclitaxel [3]. Interestingly, the Nobel Prize in Medicine 2015 was awarded for artemisinin—the active ingredient of the medicinal herb ‘sweet wormwood’—which is an effective anti-malarial therapy [4]. 

Mushrooms have long been regarded as a healthy source of nutritional value [5]. Additionally, they have emerged in recent years not only as a source of drugs, but also as adjuvants to conventional treatments. Their potential to reduce side effects related to chemotherapy or radiotherapy is of special interest. In this regard, the best investigated medicinal mushroom is *Coriolus versicolor* (L.) Quél. (1886), also known as *Trametes versicolor* (L.) Lloyd (1920), *Polyporus versicolor* (L.) Fries (1821), Turkey Tail, *Agaricus versicolor*, *Boletus versicolor*, *Polystictus versicolor*, *Poria versicolor*, Yun-Zhi (Chinese), and Kawaratake (Japanese) [6]. An ancient Chinese formulation of *Coriolus versicolor* (CV) has been used in traditional Chinese herbal medicine for over 2000 years. It is believed that CV promotes health, strength, and longevity. In traditional Chinese medicinal practice, the CV mushroom is considered useful for removing toxins, strengthening, increasing energy, improving liver and spleen function, promoting diuresis, and enhancing the immune response. It is also used to damp heat jaundice, hypochondriac pain, poor appetite, lassitude, and weakness [7].

Many studies have reported that CV has anti-oxidant, hypoglycemic, and immune-enhancing effects, and therefore, its benefits in liver disease and diabetes are expected. Indeed, in China, Japan, and the United States, CV is used as an important dietary supplement that protects the liver [8]. 

Among more than 270 recognized species of mushrooms with immunotherapeutic properties, 50 are described as non-toxic and have been tested in animal models, and 6 of these species have been studied in human cancers. The CV mushroom is the only one among these 6 species which has been studied in phase I, II, and III randomized clinical trials in stomach, colorectal, esophageal, and breast cancer patients [9]. In Japan and China, the extract from CV is prescribed routinely to radio- or chemotherapy-treated patients suffering from different types of cancer. It has been found that CV increases survival rates and improves activity of immune cells. Moreover, it counters the immunosuppressive effects of conventional anti-cancer therapies and reduces cancer treatment-related symptoms, such as fatigue, loss of appetite, vomiting, and pain, thereby improving the quality of life of cancer patients [10,11]. All these effects make CV extract a commonly used drug by many naturopathic physicians and integrative oncologists in the USA [12]. The number of publications on CV reflects great interest in the medical application of this mushroom. Over 400 publications concerning CV have been deposited in PubMed within the last 5 years.

The aim of this review is to research advances in the field of CV-induced effects that can be useful in a treatment of one of the most actual medical challenges of 21st century i.e., cancer and COVID-19.

## 2. Active Compounds in CV Extract

Since mushrooms, in contrast to animals, do not have any adaptive immune system, their chemical shield must protect them from the entire spectrum of pathogens that exist in their natural environment. The study on the composition of CV shows that it contains many compounds, such as proteins [13,14], fatty acids [15], polysaccharides [16,17], polysaccharopeptides [18,19], glucans [20,21], amino acids [15,22], vitamins [8,15], and a variety of inorganic salts [15,23]. The chemical structures of the main CV active compounds described in this review are presented in the Supplementary Materials (Figures S1–S5) [24,25,26,27]. 

### 2.1. Polysaccharopeptides

Polysaccharopeptides exert many physiological effects that are useful in the treatment of cancer, inflammation, and diabetes, such as promoting immune function and providing anti-tumor, anti-inflammation, and anti-diabetes effects [28]. Among polysaccharopeptides, polysaccharide peptide (PSP) and polysaccharide krestin (PSK) are the most studied ones for their anti-cancer and immune-enhancing properties. PSK was extracted by salting out with ammonium sulphate from the hot water extract in Japan in the 1960s and is a soluble protein-bound polysaccharide derived from the CM-101 strain of the fungus. PSP was extracted using alcohol precipitation from the hot water extract in China in the 1980s and is a polysaccharide-peptide derived from the COV-1 strain [19]. Both compounds are light or dark brown powders that are soluble and stable in water. The molecular weights of the two molecules are about 100 kDa with a respective polysaccharide-to-peptide balance of 90–10% in PSP and 60–40% in PSK. The carbohydrate moieties of each compound consist of mannose, xylose, and galactose, in addition to fructose in PSP or arabinose and rhamnose in PSK [18].

The toxicity of these compounds was tested on a variety of animals (dogs, monkeys, guinea pigs) showing no genetic and reproductive effects [29]. Importantly, neither teratogenic nor mutagenic effects of PSP have been observed in female reproductive and embryonic development in animal models [30,31]. 

### 2.2. Polysaccharides

*Coriolus versicolor* contains a high number of polysaccharides, including heteroglycan macromolecules. The main monosaccharide that builds these polysaccharides is glucose, followed by small amounts of mannose, rhamnose, glucuronic acid, and fructose [16].

Polysaccharides possess many physiological activities, such as promoting immune function and providing anti-tumor and anti-inflammatory effects [28,32]. They are effective in protecting the liver by increasing the activity of antioxidant enzymes and glutathione and the brain during cerebral ischemia reperfusion. Additionally, beneficial effects in diabetes and anti-bacterial activities have also been observed [16,33,34,35,36]. Numerous studies have shown that polysaccharides from CV can scavenge free reactive oxygen species (ROS) [37,38,39]. These effects may be useful in diseases, such as arteriosclerosis, Alzheimer’s disease, and cardiovascular and cerebrovascular diseases [40].

Among polysaccharides derived from CV, β-glucans are the principal components. Due to their non-starch and non-digestible natures, they can be utilized as dietary fibers by gut probiotic bacteria in the large intestine. Therefore, they are considered as potential prebiotics with anti-obesity properties [41,42,43]. Beta-glucans are believed to be one of the most well-established and potent derivatives of mushrooms that have anti-tumor, immunomodulatory, anti-viral, and anti-bacterial properties [21,44,45,46,47,48]. Notably, due to their confirmed complex mode of action, β-glucans are recognized as biological response modifiers. They induce epigenetic programming in innate immune cells to produce a more robust immune response and act as pathogen-associated molecular patterns (PAMPs), binding to specific pathogen recognition receptors. In consequence, innate and adaptive immune responses are induced [49,50].

### 2.3. Small Molecules

*Coriolus versicolor* contains pharmacologically active secondary metabolites belonging to small molecules. Wang et al. reported the isolation of four new spiroaxane sesquiterpenes, tramspiroins A-D, one new rosenonolactone 15,16-acetonide, and the known drimane sesquiterpenes isodrimenediol and funatrol D [51]. Moreover, Janjušević et al. identified 35 phenolic compounds belonging to the flavonoid (flavones, flavonols, flavanone, flavanols, biflavonoids, isoflavonoids) and hydroxy cinnamic acids, which exhibit anti-radical and acetylcholinesterase inhibitory properties. Therefore, CV extract can be eventually used as drug-like compounds or food supplements in the treatment of Alzheimer’s disease [52]. 

Among small molecules present in the CV extract, Yang et al. isolated a compound of about 10 kDa molecular weight named as a small peptide of *Coriolus versicolor* (SMCV). This compound inhibits in vitro proliferation of various human cancer cells, such as gastric cancer, leukemia, hepatoma and colon cancer, more significantly than PSP or PSK. Moreover, pre-treatment of SPCV decreases the proliferation of tumor cells in mouse and has an immunostimulating effect manifested by increase in white blood cells and IgG levels [53]. 

Another small molecule purified by Kuan et al. from CV is a 12-kDa non-glycosylated protein comprising 139 amino acids, including an 18-amino acids signal peptide. This protein, called YZP, has the ability to induce an increase in interleukin (IL) 10 secretion by B lymphocytes and suppress the production of pro-inflammatory cytokines by lipopolysaccharide (LPS)-activated macrophages [14]. 

Recently, He et al. characterized a novel 12-kDa protein named musarin. This protein shows significant growth inhibition on multiple human colorectal cancer cell lines in vitro. In the animal model, oral ingestion of musarin significantly inhibits tumor colorectal development at the similar level to gefitinib (a tyrosine kinase inhibitor used in oncology), but with a lower number of side effects [54]. 

## 3. Effect of CV on Immune Cell Properties

The analysis of the literature has shown that mushrooms contain many compounds that can activate the adaptive and innate immune system. The immunomodulating effects of CV have been studied intensively in in vitro and in vivo models. Clinical trials on this subject are also in progress [28,29,55].

### 3.1. Macrophages

Studies have shown that macrophages are one of the main target cells of CV extract. In vitro experiments demonstrated the direct effect of β-glucans and PSP derived from CV on the activation of macrophages, which is manifested by the increased expression of inducible nitric oxide synthase (iNOS) and production of reactive nitrogen intermediates and reactive oxygen intermediates [48,56].

The best reported immunomodulatory effect of CV is induction of cytokine productions. The water-extracted CV compounds, such as PSP and PSK, stimulate in vitro the secretion of IL-1β, IL-6, and tumor necrosis factor α (TNF-α) in different macrophage lines [57,58,59]. Moreover, the peritoneal macrophages isolated from the PSP-treated mice exhibit also increased release of prostaglandin E2 [58]. 

Considerable levels of research have been devoted to understanding how active compounds of CV mushroom interact with macrophages. Several studies have shown that the effect of the whole CV extract as well as PSP and PSK is mediated through Toll-like receptor (TLR) 4 signaling pathway, including the induction of nuclear factor κB (NF-κB) [57,59,60] and TRAF6 transcription, phosphorylation of c-Jun (a component of the transcription factor called activator protein 1 (AP-1)) [59,60], and increased expression CD14 glycoprotein (co-receptor especially required for LPS recognition by TLR4) [57]. It has been also shown that the whole CV extract induces production of cytokines, which is mediated by the phosphoinositide 3-kinase pathway [57].

In addition to TLR4, TLR2 and dectin-1 receptors are also involved in CV recognition by macrophages. Quayle et al. demonstrated that during stimulation of macrophages with PSK, a β-glucan fraction is recognized by the dectin-1 receptor, whereas lipid fraction towards TLR2 [47]. The ability of PSK to induce TNF-α production by macrophage as a result of TLR2 activation was also observed by Coy et al. [61]. Moreover, the dectin-1 signaling pathway, triggered by β-glucans isolated from CV, elicits TNF-α, nitric oxide and iNOS production leading to activation of macrophages toward phagocytosis [45,46]. Induction of phagocytosis in macrophages upon stimulation with β-glucans is also related to the increased expression of the scavenger receptor B1 (SR-B1) [20].

### 3.2. Peripheral Blood Mononuclear Cells

Numerous studies have shown that the CV extract affects all populations of peripheral blood mononuclear cells (PBMCs) as well as single cell population. One of the most documented effects of CV compounds (i.e., PSP and polysaccharides) and whole CV extract on PBMCs is the increased proliferation response. This mitogenic effect has been observed for human and rat PBMCs [62,63], human and rat lymphocytes [64,65], murine splenic lymphocytes [66], murine B lymphocytes [17] and human monocytes [67]. 

Numerous studies have shown that CV compounds, such as polysaccharopeptides and both aqueous and solid fractions of the CV mycelium, stimulate PBMCs to the secretion of predominantly pro-inflammatory cytokines. The CV extract-induced production of IL-1β, IL-2, IL-6, IL-12, and TNF-α was demonstrated in rat PBMCs [63], rat lymphocytes [65,68], and murine splenic lymphocytes [66]. Similarly, it was also reported that human PBMCs derived from healthy donors stimulated with polysaccharopeptides derived from CV secrete TNF-α [62], IL-1α, IL-2, IL-6, IL-8, IL-10, macrophage inflammatory protein 1 (MIP-1), granulocyte colony-stimulating factor (G-CSF), granulocyte-macrophage colony-stimulating factor (GM-CSF) [69,70], and interferon-γ (IFN-γ) [70,71]. Interestingly, human PBMCs isolated from breast cancer patients also exhibit an increased expression of TNF-α, IL-6, and IL-12 in response to PSP [71].

**Figure 1:**
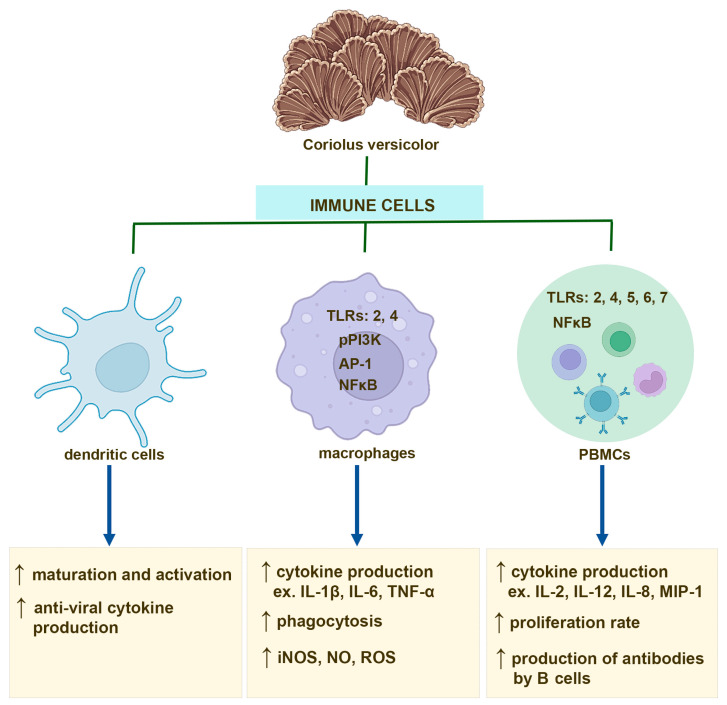
Immunostimulatory effects of the compounds derived from *Coriolus versicolor* mushroom on dendritic cells, macrophages, and peripheral blood mononuclear cells (PBMCs), including monocytes and B and T cells.

Several studies have shown that the effect of CV compounds on PBMC activation is mainly mediated through TLRs. Human PBMCs isolated from healthy donors and treated with PSP show upregulated expression of TLR4, TLR5, TLR6, and TLR7 as well as increased levels of multiple key molecules of TLR signaling pathway, such as TRAM, TRIF, and TRAF6 [69]. Interestingly, PSP also activates the TLR4-TIRAP/MAL-MyD88 signaling pathway in PBMCs derived from breast cancer patients [71]. The polysaccharides from CV exert immunoregulatory effects on B cells also via TLR4 involvement, and in consequence, inducing activation of the mitogen-activated protein kinase (MAPK) and NF-κB signaling pathways [17]. In contrast, PSK activates NK cells and monocytes to produce cytokine through binding to TLR2 [72,73]. Moreover, it has been also reported that CV compounds, including polysaccharides and PSK, are able to enhance antibody production in B cells after binding to the B cell receptor [17,74] (Figure 1).

## 4. Effect of CV Extract on Viral Infections

Among the bioactive properties of compounds derived from CV, their anti-viral activity against numerous viruses is well described. The high therapeutic index of CV extract was discovered against herpes simplex virus (HSV) type 1 and HSV type 2 in the experiments conducted on the kidney epithelial cells in vitro (EC_50_ = 77 µg/mL measured for the both HSV types) [75]. Liu et al. revealed that PSK can inhibit Epstein–Barr virus (EBV)-infected B and T cells and activate natural killer (NK) cells [76]. It has also been reported that PSP has an inhibitory effect against human immunodeficiency virus (HIV) type l reverse transcriptase and protease that are two enzymes of paramount importance to the life cycle of HIV (IC_50_ = 150 μg/mL and IC_50_ = 6.25 μg/mL measured for the interaction between HIV-1 gp120 and immobilized CD4 receptor and for the potent inhibition of recombinant HIV-1 reverse transcriptase, respectively) [77]. Rodriguez-Valentín et al. observed that PSP exerts an anti-HIV activity mediated by TLR4 and promotes the upregulation of specific anti-viral chemokines (RANTES, MIP-1) and stromal cell-derived factor 1 (SDF-1α)) known to block HIV-1 co-receptors [78]. Furthermore, oral administration of β-glucans from CV improves survival and reduces lung viral titers and weight loss in chickens and mice infected with the influenza virus [21]. CV-based vaginal gel is also available for treating women with cervical uterine high-risk human papillomavirus (HPV) infection [79,80].

Since CV compounds possess anti-viral properties against numerous viruses, it is likely that bioactive metabolites from CV might be considered as an anti-viral option against the novel coronavirus SARS-CoV-2. SARS-CoV-2 belongs to the family of coronaviruses that contains positive-sense single-stranded RNA [81]. The main viral protease, which plays an essential role in the viral life cycle, has been proposed as a key therapeutic target for drug development against coronavirus [82,83]. Since there are no effective anti-SARS-CoV-2 drugs, the natural products isolated from CV can be considered for the prevention and treatment of COVID-19.

Hetland et al. believe that CV may be utilized directly against SARS-CoV-2 infection as well as to prevent the immunological overreaction and harmful inflammation associated with COVID-19 [84]. According to Saxe, who is leading the MACH-19 (Mushrooms and Chinese Herbs for COVID-19) ongoing clinical trials approved by the Food and Drug Administration (FDA), the combination of CV with another fungus–agarikon (*Fomitopsis officinalis*) offers physiologically plausible immunomodulating capabilities against SARS-CoV-2 through the interaction with T lymphocyte receptors [85]. Rangsinth et al. examined 36 mushroom-derived bioactive compounds that potentially serve as the inhibitors of SARS-CoV-2 main protease. Indeed, 25 of 36 candidate compounds displayed the potential to inhibit this main viral protease. The most promising seems to be a betulinic acid derived from CV [86].

It is well established that COVID-19 is characterized by noticeably high concentrations of pro-inflammatory factors, such as IL-1, IL-2, IL-6, IL-8, TNF-α, monocyte chemoattractant protein-1 (MCP-1), G-CSF, GM-CSF, and many others [87]. Uncontrolled production of pro-inflammatory cytokines leads to cytokine storm in the lungs, which is initiated by the binding of the SARS-CoV-2 virus to the TLRs [88]. High levels of pro-inflammatory factors along with oxidative stress in patients with COVID-19 lead to fatal effects, such as acute respiratory distress syndrome (ARDS), pulmonary fibrosis, and death [87]. Zhang et al. demonstrated that anti-inflammatory therapy, including suppression of pro-inflammatory cytokine production, might have a therapeutic effect on viral diseases [89]. Numerous in vitro studies revealed the anti-inflammatory effects of both whole CV extract and its compounds, i.e., polysaccharopeptides, and proteins on PBMCs [63], B cells [14] and macrophages [14,57,90]. The anti-inflammatory properties of CV extract are associated, among others, with its ability to block the physical associations of pro-inflammatory factor, such as LPS with the specific receptors on immune cells (e.g., TLR4 or CD14 receptor) and decreasing the expression of these receptors. In consequence, a downregulation of NF-κB activity and pro-inflammatory cytokine production has been observed [57,90,91]. As an anti-inflammatory agent, CV extract has shown benefit in experimental animal models of osteoarthritis [35], inflammatory bowel disease [92], and traumatic brain injury [93].

Besides anti-inflammatory effects, the active compounds from CV, such as polysaccharides and protein-bound polysaccharides, also exhibit anti-oxidant properties by inducing the radical scavenging activity of superoxide dismutase and glutathione peroxidase, which was confirmed in vitro [39,94] and in vivo [37,95,96].

Published data indicate that active compounds of CV extract have the ability to inhibit inflammation and oxidative stress that is involved in the severe course of COVID-19. Furthermore, since natural products derived from CV show high efficiency in the treatment of many viruses, such as HIV, HPV, HSV, EBV, and influenza, the efficiency of CV compounds in the treatment of COVID-19 should be further investigated. 

**Figure 2:**
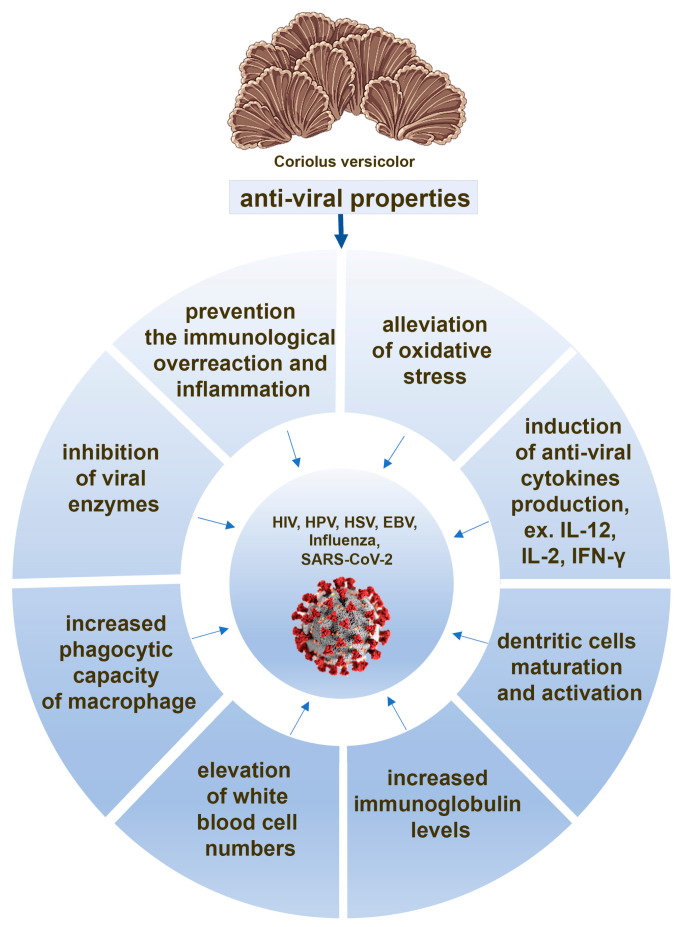
The potential anti-viral actions of the compounds derived from *Coriolus versicolor* mushroom.

There are evidences that, in response to the both whole CV extract and its polysaccharopeptides treatment, immune cells produce cytokines with anti-viral properties, such as IL-12 [66,71,97,98], IFN-γ [66,70,99], and IL-2 [90,98]. The role of these cytokines in the treatment of COVID-19 is widely discussed, showing that IFN-γ is key for restraining SARS-CoV-2 infection [100]. IL-12 is required to maintain NK cell numbers in the early phase of SARS-CoV-2 infection [101] and IL-2 deficiency appears leading to serious effects, such as weak response for our immune system against SARS-CoV-2 [102]. All these findings indicate that the ability of CV compounds to induce anti-viral cytokine production by immune cells can be considered as a potential mechanism of its action against SARS-CoV-2 (Figure 2).

The compounds from CV, such as protein-bound polysaccharides, also induce dendritic cell maturation as well as anti-viral cytokine production by activated dendritic cells [97,103,104] (Figure 1), and they have the ability to enlarge draining lymph nodes with the higher number of activated dendritic cells [97]. This adjuvant-like activity of CV compounds may have potential therapeutic value in the preparation of a more effective COVID-19 vaccine. It is also an important issue since dendritic cells have an essential role in defending against SARS-CoV-2 infection [105,106,107] (Figure 2).

## 5. Effects of CV on Cancer Cells

Nowadays, chemotherapy, hormonotherapy, and targeted therapy are regarded as one of the most promising cancer systemic treatment approaches [108,109]. However, oncogene mutations, epigenetic changes, or changes within the tumor microenvironment may, among others, trigger a strong resistance of cancer cells to various modern anticancer drugs, resulting in increased tumor cells invasion and metastases [108,110]. Therefore, for these tumors, additional treatment potentiating inhibition of their proliferation and progression may increase the survival time of patients. 

### 5.1. CV Activity in Combined Anti-Cancer Treatment

**Figure 3:**
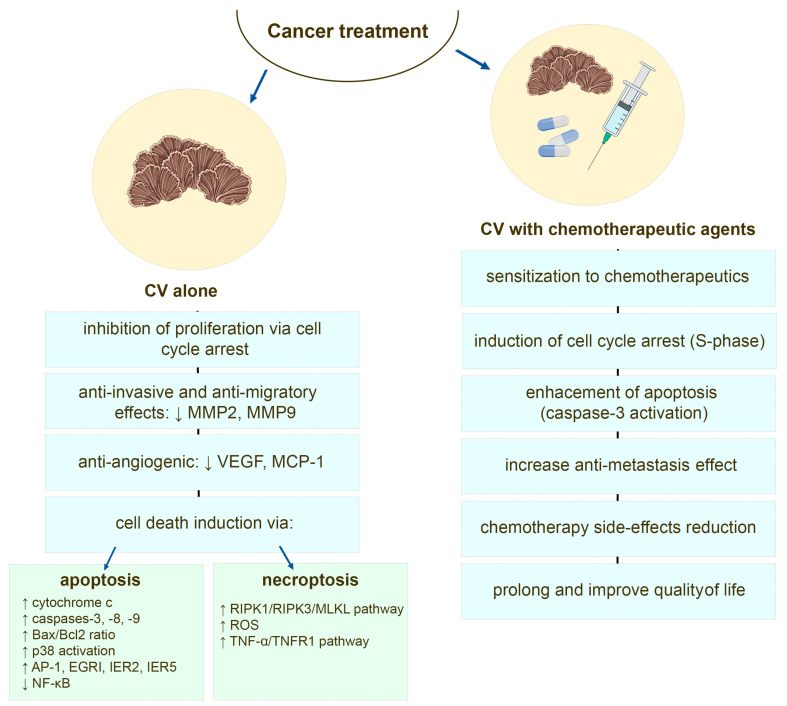
The potential anti-cancer mechanisms of the compounds derived from *Coriolus versicolor* mushroom.

There are many studies that show that bioactive compounds of CV mushroom sensitize cancer cells towards the cytotoxic effects of chemotherapeutic agents [24,111,112,113] and additional drugs used in cancer therapy [114]. Protein-bound polysaccharides from CV enhance the apoptotic machinery induced by doxorubicin and etoposide in estrogen receptor (ER) negative human breast cancer [24] and leukemia cells, where this effect is associated with an induction of S-phase cell cycle arrest and caspase 3 activation [111,115]. The PSP pre-treatment increases also the response of human leukemia cells to camptothecin (CPT), where likewise by induction of cell cycle arrest in the DNA synthesis phase, PSP sensitizes the cancer cells to undergo apoptosis induced by CPT [116]. The combination therapy of PSK and docetaxel, examined in murine model of human prostate cancer, revealed that CV components augmented tumor regression and apoptosis of cancer cells compared to the activity of this chemotherapeutic agent alone [113]. Studies performed on other animal cancer models also described PSK-boosted effects of docetaxel-induced tumor cell apoptosis [117,118]. A recent report of the study performed using intratibial breast cancer murine model also demonstrated that CV alone is effective in decreasing tumor progression, whereas in combination with zoledronic acid (ZOL), which is used in adjuvant therapy for breast cancer [119], it shows significant anti-tumor, anti-metastasis, and anti-osteolytic effects [114] (Figure 3). 

### 5.2. CV as Monotherapy for Cancer Treatment

There are plenty in vitro and in vivo studies demonstrating that CV compounds, among them PSP and PSK, besides cancer cells sensitization to various chemotherapeutic agents, can also induce tumoricidal effects [65,91,98,115,120,121,122,123,124,125,126,127,128,129,130,131,132,133,134] and have inhibitory effect on tumor growth and metastasis in animal models [66,98,120,135,136]. These effects are associated with reduced proliferation mainly via cell cycle arrest [120,123,124,131,137,138] and induction of different mode of cancer cells death, such as apoptosis, necrosis, or necroptosis [120,121,122,124,125,127,128,133,134,136]. 

In the literature, discrepancies of CV outcomes towards cancer cells are easy to observe. This may result from the preparation and extraction methods of whole CV extract, the use of its single isolated compound as well as its concentration and cancer cell type [139] (Table 1). However, direct inhibition of tumor cell proliferation induced by CV extract and its compounds, such as PSK, PSP, and CV peptide, has been reported for leukemia [134,140], breast cancer [65,68,91], melanoma [120,128], colon cancer [129], and human esophageal carcinoma [131]. The molecular mechanism of this inhibition is still under constant investigation. The disruption of CV-treated cancer cell cycle progression and arrest at G0 phase [138], G0/G1 phase [120,124,131,137], or G1/S [111,112,123] and G2/M [123] phases have been reported. The observed anti-proliferative and cytotoxic effects of CV towards cancer cells are comparable to those induced by chemotherapeutic agents and are associated with oxidative stress that induces cancer cell death [91,127,128]. Pawlikowska et al. have found that CV-induced ROS generation trigger non-pigmented melanoma cells death [128], whereas Jędrzejewski et al. have also confirmed the ROS-dependent cytotoxic activity against breast cancer cells in the pro-inflammatory environment created by LPS [91]. The in vitro and in vivo molecular studies revealed that CV extract acting alone can induce cancer cell death through different cell death modalities. The apoptotic machinery is triggered by CV components in leukemia [115,121,125,134,136], breast cancer [98,122,124], and human esophageal carcinoma cells [131]. The components of CV extract are shown to induce mitochondria-mediated apoptosis pathway, since the release of cytochrome c, activation of caspases (-3, -8, and -9), and upregulation of the expression of Bax with a concomitant downregulation of Bcl-2 is observed [115,123,136]. The study of Ho et al. also implicates that the p53 protein might differentially act as a major upstream transcriptional apoptosis activator in different cancer cell types, among them breast cancer ones [122]. Additionally, Hirahara et al. have proven that apart from caspase-3 triggering, the activation of p38 MAPK signaling cascade is involved in the PSK-induced apoptosis [134]. The upregulation of early transcription factors such as AP-1, EGRI, IER2, and IER5, and the downregulation of NF-κB transcription pathways were also found to be involved in the PSP-mediated apoptosis [133].

Besides apoptosis, induction of necrosis as a model of cell death by CV extract has also been observed [120]. The CV-stimulated melanoma cells, which are known to be relatively resistant to drug induced apoptosis [141], were found to possess both apoptotic and necrotic tumor cell death features [120]. More recent analysis also revealed that CV-induced cell death of melanoma cells is regulated by receptor-interacting protein-1 (RIPK1) and ROS, and that this process is modified by melanin content in melanoma cells [128]. The CV extract has been also reported to induce RIPK1/RIPK3/MLKL-mediated necroptosis in non-pigmented melanoma cells and depigmented (with suppressed melanogenesis) melanoma cells, since co-treatment of the cells with necroptosis inhibitors abrogated the CV-induced cell death [126,127]. The necroptotic cell death mediated by activation of TNF-α/TNFR1 pathway was also observed in CV-stimulated ER-positive breast cancer cells [126]. 

The extract derived from CV, besides anti-proliferative and cytotoxic activity, also has anti-migratory and anti-invasive potentials against numerous tumor cells, including triple-negative breast cancer [98,142], estrogen receptor (ER)-positive breast cancer [91], colon cancer [129,130], pancreatic and gastric cancer [143], and melanoma [144]. The inhibition of the invasive activity of human tumor cells mediated via suppression of matrix metalloproteinase (MMP) activates, especially those of MMP-2 and MMP-9, is postulated [98,129,130,143]. Recently, Yang and co-workers revealed that CV extract and its bioactive molecules (i.e., SMCV) may reduce cancer cell invasion directly or indirectly through the suppression of TNF-α induced MMP-3 production by inactivating the p38 MAPK pathway in malignant cells [132] (Figure 3).

**Table 1:** IC_50_ values of different cancer cell lines stimulated with the whole CV extract or its compounds.

CV Compound	Cancer Cell Line	IC_50_ Value (µg/mL)	References
ethanol-water whole extract	BT-20 breast cancer cells MDA-MB-231 breast cancer cells MCF-7 breast cancer cells T-47D breast cancer	>800 514.0 271.7 233.3	Ho et al., 2005 [122]
aqueous ethanol whole extract	HL-60 leukemia cells	150.6	Ho et al., 2006 [136]
methanol whole extract	B16 melanoma cells	200	Harhaji et al., 2010 [120]
musarin (protein)	T84 colorectal cancer cells	1.8	He et al., 2021 [54]
α-glucans and β-glucans	LoVo colon carcinoma cells	224.0	Roca-Lema et al., 2019 [129]
aqueous ethanol whole extract	HL-60 leukemia cells B-cell lymphoma (Raji) NB-4 leukemia cells	147.3 253.8 269.3	Lau et al., 2004 [125]
small peptide of *Coriolus versicolor* (SPCV)	HL-60 leukemia cells LS174-T colony cancer cells SMMU-7721 hepatoma cells SCG-7901 stomach cancer cells	30.0 142.0 138.0 323.0	Yang et al., 1992 [53]
*Coriolus versicolor* polysaccharide (CVP)	7703 hepatocellular carcinoma cells BCap3 breast cancer cells T-47D breast cancer cells MCF-7 breast cancer cells ZR75-30 breast cancer cells	18.4 14.4 9.3 39.3 34.6	Zhou et al., 2007 [145]
*Coriolus versicolor* polysaccharide (CVP)	7703 hepatocellular carcinoma cells	4.25	Cai et al., 2010 [146]
PSK	MCF-7 breast cancer cells	200	Aoyagi et al., 1997 [147]
PSK	hepatocellular carcinoma H4-II-E cells human ovarian cancer cells	1.5 0.33	Kobayashi et al., 1994 [148]
ethanol whole extract	cervix adenocarcinoma HeLa cells colon carcinoma LS174 cells lung adenocarcinoma A549 cells	42.4 86.1 65.6	Knežević et al., 2018 [149]

### 5.3. CV Extract Affects Cancer Angiogenesis

The importance of angiogenesis in cancer progression is well established [150]. The inhibition of this multi-stage process, defined as the new and abnormal blood vessels network development, involves endothelial cells proliferation and organization, migration as well as invasion [116,151]. Since contemporarily used anti-angiogenic drugs, based on the blockade of vascular endothelial growth factor (VEGF) signaling pathway, so far have not displayed a clinically significant benefit either as monotherapy or as a combined anticancer treatment, other methods of endothelial cell inhibition are still sought as valuable new approach to cancer therapy [152,153]. Apart from the improvement of these strategies, several other anti-angiogenic approaches are currently being investigated, such as the use of natural non-toxic phytochemicals as anti-angiogenic agents in cancer disease [154,155]. More than three decades ago, Kanoh et al. and subsequently Wada et al., using murine model of angiogenesis and a rat cornea assay as an in vivo model of fibroblast growth factor (bFGF)-induced angiogenesis, respectively, revealed that PSK from CV suppresses tumor-induced capillary vessel formation [135,156]. Further in vitro analysis showed that PSK inhibits the proliferation of endothelial cells in the presence or absence of basic bFGF as well as suppresses the bFGF-induced MAPK kinase phosphorylation [156]. The evidence of anti-angiogenic activities of PSP were also confirmed in in vivo sarcoma tumor-bearing mouse model [157], where PSP-treated tumor displayed a vasculature of few blood vessels of much less density than controls. This anti-angiogenic effect was mediated via suppression of VEGF gene expression [157]. A recent report revealed that CV compounds have the ability to decrease the release of pro-angiogenic cytokines, such as IL-6 and IL-8, in the chronic inflammatory environment, where this effect is accompanied by a decreased expression of TLR4 and phospho-IκB [91]. The CV effect on the tumor-associated macrophages (TAMs), which are the major source of angiogenic factors boosting the angiogenic switch [158] was proven in co-culture studies, where the CV-induced disruption in the crosstalk between breast cancer cells and macrophages has been reported [142]. By altering TAMs from M2 to M1 subtype, the protein-bound polysaccharides can indirectly reduce the amount of MMPs in the tumor microenvironment. The inhibitory effect on the production of angiogenesis-related factors (MCP-1 and VEGF) in macrophages was also observed [142] (Figure 3).

## 6. Significance of Fever for Cancer and Infectious Disease: Potential Utility of CV Extract

Fever is caused by the immune contact with PAMPs that is sensed by TLRs. The existence of a large number of TLRs enables the innate immune system to detect various PAMPs, including those of bacterial and viral origin. Stimulation of TLRs by PAMPs induces activation of signal transduction cascades. This cascade leads to translocation of NF-κB to the nucleus and activation of interferon regulatory factors 3/7 (IRF3/7) and/or activator AP-1, which cooperate to induce transcription of various cytokines including IFNs (IFNα/β) to counteract infections [159,160]. Thus, fever is not only an increase in body temperature, it is a mechanism that triggers production of many factors that are involved in immune response against many dangers, including viruses and cancer cells.

Fever is one of the main presenting symptoms of COVID-19 [161], but little public attention has been given to it as a defense mechanism. This issue was addressed in a review paper highlighting that using non-steroidal anti-inflammatory drugs (NSAIDs) to inhibit SARS-CoV-2 fever in the early stages of infection may contribute to worse outcome [162]. Since fever is a well-recognized immunostimulant, numerous reports on infections show improved survival in organisms that develop fever [161,163]. This effect is a result of fever-triggered activation of the anti-viral response [164]. Additionally, a decrease in replication of various viruses [161], including SARS-CoV-2 [165,166], has been observed in febrile temperatures. Thus, an effectively functioning immune system utilizes fever during infections. Interestingly, cancer patients reveal a history of fewer fevers during infectious diseases than people without cancer [167]. It seems unfavorable for them, since it is known that high fever is inversely related to cancer incidence [168,169]. To date, it is not known what makes it impossible to generate fever in cancer patients in response to pyrogens. Therefore, it seems necessary to find agents that can restore the ability to induce fever in cancer patients. 

The analysis of CV-induced cytokines in animals showed an increase in the production of pro-inflammatory cytokines, such as IL-6, and TNF-α, typically involved in fever induction [170]. However, it has been found that CV extract alone (in a dose of 50–200 mg/kg) does not provoke fever, but induces a significant decrease in body temperature [171]. 

Different effects were observed in models of inflammation, when CV was administered together with endotoxin, such as LPS. Pre-injection of CV extract before LPS administration extended the duration of endotoxin fever in rats. This phenomenon was accompanied by a significant elevation of IL-6 level in plasma and pre-treatment of these rats with anti-IL-6 neutralizing antibody prevented this prolongation of endotoxin fever [170].

If an organism is exposed to endotoxin several times, a phenomenon called endotoxin tolerance develops. In this state, a decrease in the expression of pro-inflammatory cytokines is observed. In agreement, it was demonstrated that PBMCs isolated from LPS-tolerant rats produced significantly less IL-6 than the cells isolated from control animals in response to LPS stimulation in vitro. Importantly, the injection of CV extract partially prevented endotoxin tolerance development, and therefore, febrile increase in body temperature accompanied with an increased level of IL-6 was observed [172]. 

Pawlikowska et al. investigated whether fever-range temperatures affect CV action. It has been found that blood-derived lymphocytes cultured in fever range-hyperthermia (39.5 °C) display remarkable decrease in cell proliferation induced by protein-bound polysaccharides isolated from CV extract. This effect corresponded to the downregulation of mRNA expression of pro-inflammatory cytokines, such as IL-1β and IL-6. Furthermore, expression of these cytokines was also downregulated compared to cells cultured at 37 °C and stimulated with CV extract alone. Interestingly, in the cells which underwent combined treatment compared to ones stimulated with CV extract alone, the mRNA of TNF-α was slightly increased [68].

## 7. Clinical Trials

Despite PSK produced by Kureha Chemicals (Iwaki, Japan) and PSP being introduced on the market [173] in Japan in 1977 and China in 1987 [29], its clinical use in Europe and USA is still not approved by the European Medicines Agency (EMA) and FDA. Furthermore, in addition to in vitro studies and those using animal models on the role of CV administration during cancer and infectious diseases, only few clinical trials were performed or are ongoing. 

Torkelson et al. observed that in breast cancer patients treated with radiotherapy, the administration of CV extract increases NK cytotoxic function and lymphocyte counts [12]. In the other clinical trials, the breast cancer patients who have taken PSP and Danshen (another herbal derivative from *Salvia miltiorrhiza*) respond to the treatment with the increase in T-helper lymphocytes (CD4+) and B lymphocytes number [174]. An elevation in leukocyte and neutrophil counts, as well as serum IgG and IgM levels, were also observed in non-small cell lung cancer patients treated with PSP [175]. In addition, Bao et al. noticed lower lymphopenia during radiotherapy of patients with nasopharyngeal carcinoma after the administration of combination of PSP-Danshen [176]. 

Clinical trials conducted in gastric, lung, or colorectal cancer patients have shown that simultaneous PSP/PSK treatment along with chemotherapy boosts their immune function, including NK cell activity [13,177,178]. Both molecules have been reported to possess a beneficial effect on extending the survival rate in cancer patients [13]. Moreover, trials involving patients with advanced hepatocellular carcinoma with malfunction of the liver confirmed the positive effect of daily consumption of CV capsules on their quality of life [179]. 

Polysaccharides from CV are also promising in the treatment of hepatitis B as well as HPV [29,180]. Serrano et al. demonstrated that Papilocare (Procare Health, Valencia, Spain), which is a CV-based vaginal gel has given a better clinical benefit than the conventional treatment in clinical practice for high-risk HPV patients in terms of its efficacy to treat HPV-related cervical lesions and to clear all HPV strains after a single 6-month period of use [181]. Another clinical trial, conducted by Scuto et al., showed that CV supplementation minimizes consequences associated with neurodegeneration, neuroinflammation, and oxidative stress of cochleovestibular system pathologies, including Meniere’s disease [182]. Anti-oxidative properties of CV were also noticed in clinical trials involving patients with breast cancer [95,96]. In addition, polysaccharopeptides from CV have been clinically tested as a prebiotic on the gut microbiota of healthy volunteers [42] and in patients with inflammatory bowel diseases (CV powder as an ingredient of Mycodigest supplement [183]).

Furthermore, in a clinical trial database approved by FDA [183], there are two ongoing clinical trials related to COVID-19. One of them concerns testing mushroom-based products as a drug for COVID-19. The influence of 14-days consumption of CV or *Fomitopsis officinalis* (Fo) capsules on recovery patients with COVID-19-positive test with mild-to-moderate symptoms, who do not require hospitalization will be assessed. Taking into account data on the immunomodulating and immunostimulating properties of mushroom extract, the scientists from the University of California, decided to use CV or Fo capsule as an adjunct to COVID-19 vaccination. The randomized, double-blind clinical trial to evaluate the effect of dietary supplementation of CV or Fo capsule on titration of antibody and on mild side-effects after vaccination is planned. 

Apart from clinical trials conducted on cancer or infectious patients, the beneficial immunostimulating effect of CV extract was also confirmed in healthy volunteers in a randomized, double-blind clinical trial conducted by Wong and co-workers [184]. The elevation of PBMC gene expression of IL-2 receptor, increase in absolute counts of T helper cells and ratio of T helper/T suppressor and cytotoxic cells as well as enhancement of ex vivo production of IFN-γ by activated PBMCs have been observed. Importantly, CV consumption had no adverse effects on liver or renal functions, and therefore, it can be considered beneficial for the immunological defense of healthy subjects [184].

## 8. Conclusions

Since the current most common challenges in medicine, among which COVID-19 and cancer, are still fatal, there is an urgent need for finding new remedies. Medicinal mushrooms have been always an important source for the discovery of new therapeutics for human diseases. An ancient Chinese formulation of CV has been used in traditional Chinese herbal medicine for over 2000 years. In recent years, scientific research into its health-promoting properties has intensified.

This mushroom shows a wide spectrum of benefits, which may be useful in combating modern medical challenges. Herein, we have presented data determining the proof of strong anti-viral, anti-inflammatory, anti-oxidative, and immunostimulating properties of CV. Simultaneously, other reports confirmed the impressive anti-cancer response of CV extract and its compounds directed towards wide range of cancer types and revealed the molecular background of this process. By induction of different cell death modalities, such as apoptosis or necroptosis, CV extract appears to be an effective adjuvant therapy. Moreover, analysis of other reports revealed that CV also affects fever, the innate immunity mechanism beneficial for both cancer and viral infections recovery.

This review has some limitations resulting mainly from the procedures of CV preparation performed by a variety of research groups. The authors used different doses of either whole CV extract or its single compounds. Furthermore, they often used different extraction methods, which can result in inconsistent compositions of an extract, even if the same material was used. Moreover, many papers do not provide detailed information regarding the composition of the CV extract, which makes it impossible to clearly compare the results obtained by different authors. Furthermore, research showing a direct effect of CV extract in the treatment of SARS-CoV-2 are needed to clearly confirm its potential as an effective agent against COVID-19 disease.

Despite the discrepancies mentioned above, *Coriolus versicolor* belongs to standard oncologic treatment in the mainstream modern Japanese cancer system. The Western countries’ oncologists have only recently begun to turn their attention to immune potentiating therapies. Therefore, in order to proceed with clinical trials in the United States and Europe, the immunological and anti-cancer mechanisms must be well-established to justify proceeding with the prospective human clinical trials. In this review, a wide spectrum of research data showing the potential of CV in the treatment of COVID-19 and cancer diseases was presented. The dissemination of this knowledge is important to plan randomized clinical trials confirming all these beneficial effects in patients.

## Data Availability

Not applicable.

## Supplementary Materials

The following supporting information can be downloaded at: https://www.mdpi.com/article/10.3390/ijms24054864/s1.
