# Supplementary material for: COVID-19 and Cancer Diseases—The Potential of Coriolus versicolor Mushroom to Combat Global Health Challenges
Source: Int J Mol Sci. 2023 Mar 2;24(5):4864. doi: 10.3390/ijms24054864 (PMC10003402; doi:10.3390/ijms24054864)
Supplement: Supplementary file 1 [file ijms-24-04864-s001.zip › ijms-2155154-supplementary.pdf]

COVID-19 and cancer diseases – the potential of *Coriolus versicolor* mushroom to combat global health challenges

Supplementary materials – Chemical structures of the main active CV compounds.

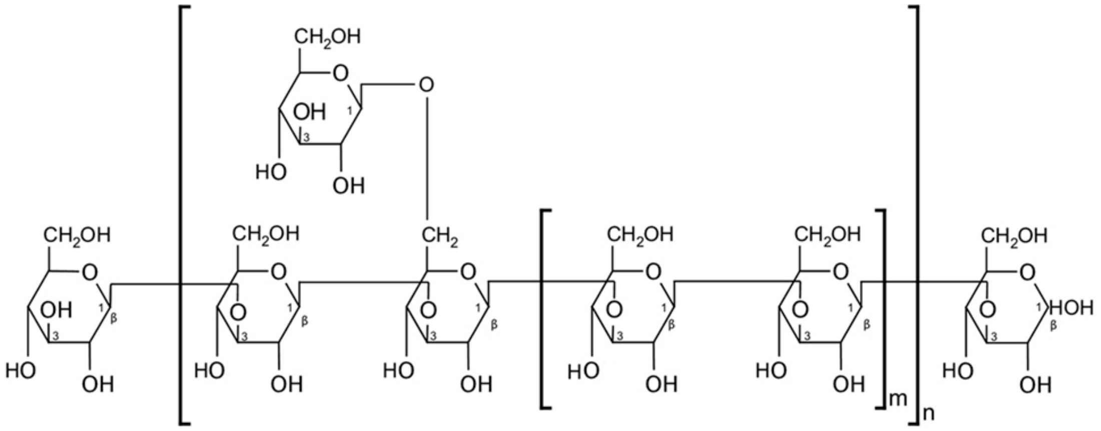

Figure S1. Polysaccharopeptide (PSP). Figure replicated from Wan et al. (24).

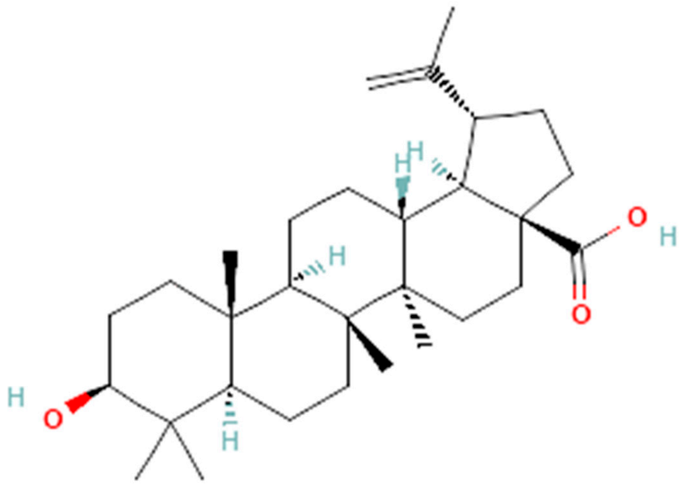

Figure S2. Betulinic acid. Figure replicated from (25).

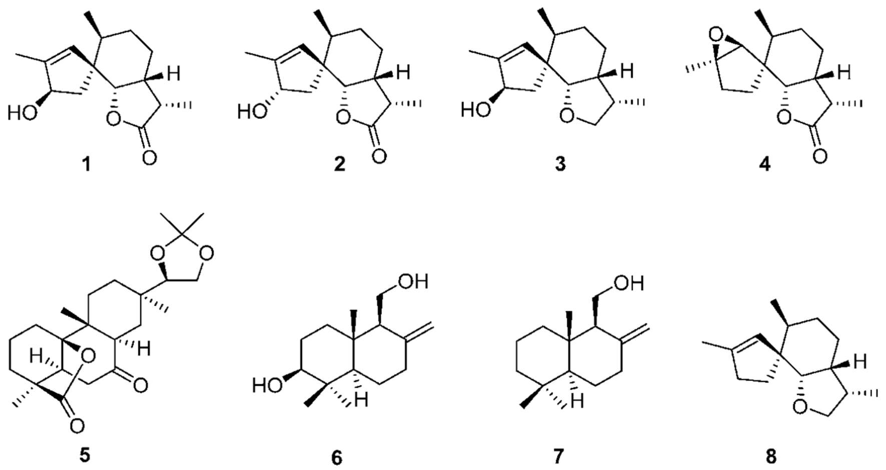

Figure S3. Terpenoids: Tramspiroins A–D (1–4), rosenonolactone 15,16-acetonide (5), and the drimane sesquiterpenes isodrimenediol (6) and funatrol D (7). Figures replicated from Habtemariam (26).

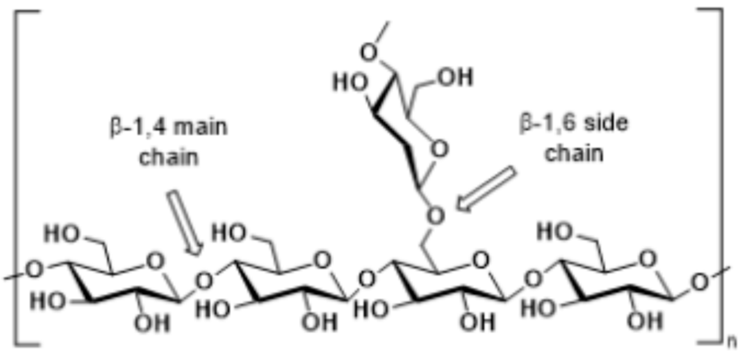

Figure S4. Polysaccharide krestin (PSK). Figure replicated from Sivanesan et al. (27).

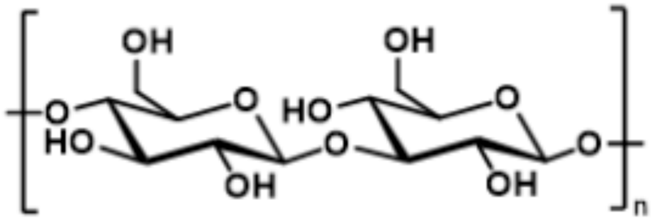

Figure S5. β-1-3-D glucan (backbone). Figure replicated from Sivanesan et al. (27).
